# The Phylogenetic Likelihood Library

**DOI:** 10.1093/sysbio/syu084

**Published:** 2014-10-30

**Authors:** T. Flouri, F. Izquierdo-Carrasco, D. Darriba, A.J. Aberer, L.-T. Nguyen, B.Q. Minh, A. Von Haeseler, A. Stamatakis

**Affiliations:** ^1^Heidelberg Institute for Theoretical Studies, Heidelberg Institute, 69118 Heidelberg, Germany; ^2^Center for Integrative Bioinformatics Vienna, Max F. Perutz Laboratories, University of Vienna, Medical University of Vienna, A-1030 Vienna, Austria; ^3^Bioinformatics and Computational Biology, Faculty of Computer Science, University of Vienna, A-1090 Vienna, Austria; and ^4^Karlsruhe Institute of Technology, Institute for Theoretical Informatics, Postfach 6980, 76128 Karlsruhe, Germany;

**Keywords:** Maximum likelihood, parallel computing, phylogenetics

## Abstract

We introduce the Phylogenetic Likelihood Library (PLL), a highly optimized application programming interface for developing likelihood-based phylogenetic inference and postanalysis software. The PLL implements appropriate data structures and functions that allow users to quickly implement common, error-prone, and labor-intensive tasks, such as likelihood calculations, model parameter as well as branch length optimization, and tree space exploration. The highly optimized and parallelized implementation of the phylogenetic likelihood function and a thorough documentation provide a framework for rapid development of scalable parallel phylogenetic software. By example of two likelihood-based phylogenetic codes we show that the PLL improves the sequential performance of current software by a factor of 2–10 while requiring only 1 month of programming time for integration. We show that, when numerical scaling for preventing floating point underflow is enabled, the double precision likelihood calculations in the PLL are up to 1.9 times faster than those in BEAGLE. On an empirical DNA dataset with 2000 taxa the AVX version of PLL is 4 times faster than BEAGLE (scaling enabled and required). The PLL is available at http://www.libpll.org under the GNU General Public License (GPL).

In phylogenetics, the implementation of likelihood calculations on trees often represents the major obstacle for testing new ideas. A substantial amount of time is spent to implement common functions and data structures. This typically delays research by several months, before testing the ideas at hand. In addition, these implementations are often inefficient because of the lack of familiarity with low-level optimization and parallel programming techniques, since researchers cannot be experts in every domain.

Thus, software libraries can contribute substantially to the rapid development of applications. For instance, Bio++ (Guguen et al. 2013) is a general-purpose C++ library that has gained popularity and includes a molecular phylogenetics module, which is characterized by its ease of use and plethora of available functions. However, such general-purpose libraries do not necessarily focus on performance, and are therefore typically not suited for specific compute-intensive tasks such as likelihood calculations. In contrast, the BEAGLE (Ayres et al. 2012) library has been specifically designed to speed up likelihood-based programs such as MrBayes (Ronquist et al. 2012) and BEAST (Drummond et al. 2012), attaining over 60-fold speedups on codon data over the native implementations. However, BEAGLE only implements the “pure” likelihood calculations and does not incorporate a tree data structure. On the one hand, this facilitates integration with existing software, but on the other makes it harder to build new tools from scratch.

To bridge the gap between speed and ease of use, we developed the *Phylogenetic Likelihood Library* (PLL), a software library that offers an application programming interface for fast prototyping and deployment of high-performance likelihood-based phylogenetic software. For efficient likelihood calculations, the PLL deploys 128- and 256-bit vector instructions, and offers an Intel Xeon PHI implementation. Apart from the likelihood functions, PLL provides functions for parsing input files, exploring tree space, and for checkpointing. It supports partitioned analyses and one can combine distinct data types to conduct joint likelihood computations on concatenated datasets containing binary, amino acid, and DNA data. Finally, the PLL supports multi-core systems via POSIX Threads and usage of Massively Parallel Processors via Message Passing Interface (MPI). The underlying complexity of the parallel code is transparent to the user.

## Application Programming Interface

### Overview

**Figure 1.**
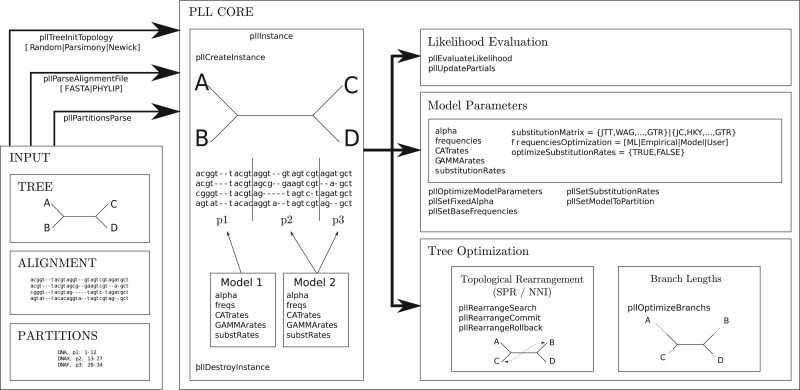
Architecture overview of the PLL.

The PLL functions are organized into two groups that handle different types of computations: 1) parsers for trees, multiple sequence alignments (MSAs), and partitioned model file formats; and 2) functions for tree space exploration, model parameter optimization, likelihood evaluation, and ancestral state reconstruction. The PLL offers an internal data structure for representing unrooted bifurcating trees, which is particularly useful for developing novel application codes from scratch. Optimized likelihood evaluation functions and topological rearrangement operations such as *Subtree Pruning and Regrafting* (SPR), *Tree Bisection and Reconnection* (TBR), and *Nearest Neighbor Interchange* (NNI) for exploring tree space are also available. In addition, PLL also implements numerical optimization functions such as the Newton-Raphson method for branch lengths or Brent's algorithm for other model parameters. Moreover, the PLL accommodates partitioned analyses where distinct sets of likelihood model parameters are optimized for different parts of the MSA. Finally, PLL also includes a highly optimized and vectorized parsimony implementation (Alachiotis and Stamatakis 2011) that can be used to generate parsimony starting trees or to filter promising SPR and TBR moves based on their parsimony scores. An overview over the software architecture of the PLL is provided in Figure 1.

### Usage

To load and access the library, a client program must first create a PLL instance by invoking the library function pllCreateInstance and passing an argument of type pllInstanceAttr. This mandatory argument describes key attributes of the PLL instance, such as the model of rate heterogeneity that can either be the Γ model (Yang 1994) or the per-site rate (PSR) model (Stamatakis 2006a), flags on whether to use a memory-saving technique (Izquierdo-Carrasco et al. 2011) and fast numerical scaling techniques, the number of threads to use in the PThreads version of the PLL, and a seed for the random number generator. The call returns an instance handle of type pllInstance that encapsulates all PLL core components.

Next, the client program must load an MSA and optionally a partition file, which splits the MSA into partitions and assigns an evolutionary model to each partition. PLL implements the *General Time Reversible* (GTR; Tavaré 1986) model, which can be used for nucleotide and amino acid data. Moreover, for DNA data a linkage specification for substitution rates and nucleotide frequencies can be set to generate all 203 possible GTR substitution matrices (Huelsenbeck et al. 2004), including the most common ones (e.g., JC69/F81 and K80/HKY85—see Yang 2006). Given that the GTR model is rarely used on protein data, the library implements 18 widely used empirical amino acid replacement models (see Supplementary Material available on Dryad http://dx.doi.org/10.5061/dryad.9dk62 for a complete list). In addition to fixed state frequencies (e.g., as provided by the protein models), PLL can employ empirical state frequencies or a maximum-likelihood estimate of the state frequencies. PLL also supports binary data with an option to correct for ascertainment bias as specified in the original model by Lewis (2001). An analogous ascertainment bias correction mechanism for accommodating the a priori exclusion of variable sites is also available for analyzing DNA SNPs datasets. PLL does not currently implement either time-heterogeneous models or models for among-partition rate variation.

The next step for the client program is to either pass a starting tree to the PLL or use the PLL to generate a random tree or a randomized stepwise addition order parsimony tree. The starting tree and the optional list of alignment partitions is then loaded into the PLL instance.

To calculate the likelihood of the tree, the client program calls pllEvaluateLikelihood by passing a node that is the end-point of the edge on which the virtual root shall be placed. The virtual root acts as the “direction” for the likelihood computation, indicating that the likelihood shall be computed bottom-up, from the leaves to the virtual root, with the two end-points of the selected edge being the two direct descendants of the virtual root.

The PLL offers two methods for computing the likelihood, which differ in the number of conditional likelihood vector (CLV) computations: *partial* or *full* tree traversals. This difference is associated with the CLVs storage technique, also known as views (Felsenstein 2004), at inner nodes. Instead of storing three CLVs per inner node, that is, one per outgoing edge, the PLL only stores one CLV and keeps track of the direction of the computation. When choosing an evaluation via a full traversal, the PLL (re-)computes the CLVs for all nodes, and finally calculates the likelihood of the tree topology. A full tree traversal is necessary when model parameters (e.g., the α shape parameter of the Γ distribution or substitution rates) have changed, and hence the stored CLVs become invalid. A partial traversal will only recompute the CLVs of those nodes whose direction is inconsistent with the new direction of likelihood evaluations, specified by an altered location of the virtual root. Therefore, the placement of the virtual root, even though it is irrelevant for full traversals, affects the speed of likelihood evaluations when using partial traversals. Partial traversals are useful, for instance, when applying local topological changes to a tree.

## Materials and Methods

The PLL is implemented in C and is currently available for Linux, MAC, and Windows.

### SIMD Implementations

All modern processors have the so-called single-instruction multiple-data (SIMD) capabilities. SIMD processors are capable of applying the same arithmetic operation to multiple elements of a data vector simultaneously.

SIMD instructions can be used by loading data into specific vector *registers* (storage space within the (co)processor) with a length of 128, 256, and soon 512 bits. Those registers can hold multiple, independent data values of smaller size (e.g., a 256-bit register can hold four 64-bit double-precision floating-point values). If two registers contain four values each, for instance, we can perform element-wise multiplications of the vector elements using a single instruction, instead of executing four separate instructions. Typically, Intel-based architectures employ 128-bit registers using *streaming SIMD extensions* (SSE), and 256-bit registers using *advanced vector extensions* (AVX) instructions. The AVX-2 instruction set implements some additional and more complex vector operations (fused multiply–add instructions) that can be leveraged by likelihood calculations. In the future, AVX-512 will offer 512-bit-long registers/vectors.

The phylogenetic likelihood function (PLF) typically accounts for >95% of total execution time in current state-of-the-art likelihood-based tools for phylogenetic inference (Stamatakis 2006b). Therefore, the acceleration and parallelization of the PLF is a crucial task. Except for the generic unoptimized version of the PLF, PLL comes with three optimized versions that use the SSE3, AVX, and AVX-2 instruction sets.

### PThreads and MPI Implementation

The PLL uses a master-worker paradigm to parallelize the likelihood function by splitting up the computation of the per-site entries of one CLV (associated with a single node) at a time among processes. The master process triggers likelihood calculations and orchestrates the optimization of branch lengths and model parameters. As mentioned before, the tree must be fully (re-)traversed to optimize substitution rates or the α-shape parameter. After a change in these model parameters, the master triggers a full tree traversal and every worker process independently updates its fraction of the CLV entries for each node. The worker processes are agnostic of the tree topology and alignment structure, that is, they are not required to maintain and update the current tree, and only operate on consistently enumerated CLVs that correspond to nodes in the tree.

For obtaining “good” load balance, when optimizing ML or proposing new model parameters (Bayesian Inference) for partitioned datasets, it is important to propose and evaluate these new values (e.g., α-shape values) simultaneously for all partitions to improve parallel efficiency (Stamatakis and Ott 2009). In addition, a well-balanced number of sites must be assigned to each process, and the number of distinct partitions per process (Zhang and Stamatakis 2012) needs to be minimized. Since the sites in each partition evolve under a different model, each partition incurs an additional computational overhead (e.g., calculating the transition probability matrix). Another technique for load balancing is to distribute likelihood computation to processes on a “per-node” basis. This, however, induces a substantially increased synchronization overhead due to node dependencies when computing the CLVs, particularly for imbalanced trees and trees with a relatively small number of taxa compared to the available number of processors. Furthermore, most of typical CLV updates in phylogenetics application are only partial updates, which further limits the degree of parallelism in a per-node parallelization approach. Overall, PLL implements two approaches for load balancing: 1) a cyclic distribution of the alignment sites; and 2) a monolithic partition distribution.

The cyclic distribution works better when the input alignment consists of at most as many partitions as there are processes. The second approach distributes partitions monolithically (entire partitions are assigned to one process) among processes, and hence it is important to have a considerably higher number of partitions than processes (Zhang and Stamatakis 2012).

Recently, Kobert et al. (2014) showed that this load-balancing problem is NP-hard and designed a polynomial time approximation algorithm that is guaranteed to yield a near-optimal distribution of sites and partitions to processes. The algorithm balances the number of sites among processes and at the same time minimizes the number of partitions assigned to each process. Note that, in this setting, a single partition can be split among several processes. The implementation of this new method in PLL is underway.

## Experimental Results

The PLL has successfully been integrated with two phylogenetic software packages: DPPDiv (Heath et al. 2012), a Bayesian tool for estimating divergence times on a fixed tree topology; and IQ-TREE (Minh et al. 2013), a tool for reconstructing maximum-likelihood trees and assessing branch support with an ultra-fast bootstrap approximation. In the following, we first provide a performance comparison between PLL and BEAGLE and then summarize the results for integrating and accelerating DPPDiv and IQ-TREE with the PLL.

### PLL versus BEAGLE

Here, we discuss the differences between PLL and BEAGLE and experimentally assess their performance and correctness (all necessary files and instructions for repeating the experiments are available at http://www.libpll.org/beagle-pll-experiments). For testing, we used a 4-core Intel i7-2600 multi-core system with 16 GB RAM and swapping disabled.

The main difference between PLL and BEAGLE lies in the data structure. The PLL provides (and requires) its own data structure that encapsulates the tree topology, alignment, and model parameters. In contrast to this, BEAGLE comes with no data structure, and requires the client program to map its own tree data structure to a BEAGLE instance by initializing the sequence data and defining the postorder traversal of the tree. This approach makes BEAGLE easier to integrate with existing likelihood-based software that already has a native tree data structure. In contrast to this and by using an internal tree structure, the PLL can offer 1) global branch length optimization across the entire tree, 2) topological rearrangements, and 3) model parameter optimization (e.g., substitution rates, branch lengths, or base frequencies).

Therefore, PLL is better suited when developing new software from scratch: It provides functions for handling FASTA, PHYLIP, and Newick files; explicitly supports partitioned analyses including support for load-balance, and the client application may directly use the PLL tree structure as its primary data structure and benefit from tree space exploration and branch length optimization functions.

**Figure 2.**
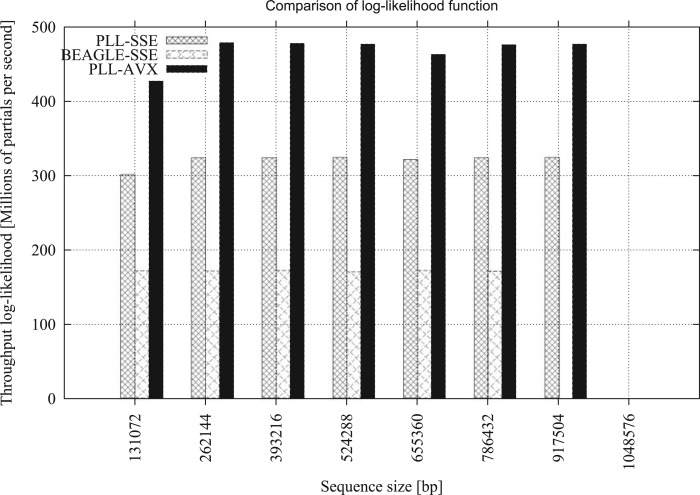
PLF performance of PLL versus BEAGLE for 100 likelihood evaluations using full tree traversals, on a dataset with 128 taxa and varying alignment length.

Both libraries implement vectorized versions of the PLF. BEAGLE comes with a double-precision SSE implementation, whereas PLL comes with double-precision implementations using SSE or AVX. To compare the PLF performance of the two libraries, we used a random tree topology of 128 taxa, and randomly generated alignments with lengths of 131,072, 262,144, 393,216, 524,288, 656,360, 786,432, 917,504 and 1,048,576 bp. We used randomly generated sequences to thoroughly study the numerical stability of the libraries. Figure 2 illustrates the performance in CLV entry updates per second. This number was obtained by executing 100 independent full tree traversals, where each iteration recomputes all CLVs from scratch. The term independent means that each full tree traversal is independent from the others, that is, none of the 100 tree traversals is allowed to reuse intermediate results (such as scaling factors) from preceding traversals. We then used the time (in seconds) it took the fastest out of these 100 tree traversals to calculate the CLV entry updates per second. The plot shows that, the SSE version of PLL is up to 1.9 times faster than the corresponding BEAGLE SSE implementation. We were only able to assess sequential AVX performance of the PLL, since sequential AVX support is not yet available in BEAGLE (see Supplementary Material available on Dryad http://dx.doi.org/10.5061/dryad.9dk62 for parallel AVX results with OpenCL in BEAGLE and details on the comparison of the two libraries). We executed 100 independent likelihood computations to eliminate the potential impact of server-side events, such as context switching or daemons, on the benchmark. The run-times of each of the 100 executions are identical with no external interference. The benchmark timings only include the core likelihood computations. All intermediate data structures for tree and alignment parsing were deallocated once the instances of PLL/BEAGLE were initialized. The order of the tree traversal (or direction of likelihood computations) was set using the BeagleOperation structure in BEAGLE and for PLL using its dedicated tree structure. On the 128-taxon alignment with a length of 917,504 bp, BEAGLE ran out of memory while the PLL was still able to process and compute the likelihood. The last alignment (1,048,576) was not computed by either library due to lack of memory. We observed that, BEAGLE requires ∼ 12% more memory than the PLL for storing the scaling factors as arrays. Scaling factors are stored more efficiently in PLL using only one scaling factor per node and per partition. We also compared the performance on an empirical DNA dataset with 2000 taxa and a length of 1251 bp from (Pattengale et al. 2009). For 1000 independent full tree traversals, BEAGLE (SSE) required 312.6 s, PLL-SSE 121.2 s, and PLL-AVX 79.1 s. The results indicate that the PLL is approximately 2.6 times faster than BEAGLE, when comparing the SSE double-precision implementations of the likelihood function with numerical scaling enabled. Thus, PLL-AVX is up to 4 times faster than the BEAGLE SSE version when analyzing empirical DNA datasets that require frequent numerical scaling operations to prevent arithmetic underflow. With numerical scaling disabled, BEAGLE is faster than PLL, since PLL always computes scaling factors by default.

Finally, PLL is ready for high-performance computing and features a POSIX threads and MPI implementation (Supplementary Material available on Dryad http://dx.doi.org/10.5061/dryad.9dk62 for parallel performance data), as well as a port for the Intel Xeon PHI coprocessor.

### DPPDiv

DPPDiv is a Bayesian tool for estimating species divergence times and lineage-specific substitution rates. Given a fixed topology, it estimates these parameters under a nonparametric mixture model. More specifically, DPPDiv uses a Dirichlet process prior to model variation of substitution rates across the branches of the tree. In evolutionary biology, divergence time estimates are widely used for analyses of historical biogeography, species diversification, and trait evolution.

DPPDiv uses *Markov Chain Monte Carlo* to sample the posterior densities of the model parameters and to estimate lineage-specific substitution rates as well as node ages. Each time the Markov chain proposes a new state, the PLF is invoked, and it needs to be computed for each branch in the tree and for each rate category. In fact, DPPDiv spends over 90% of overall execution time on PLL calculations.

To optimize the code, we integrated PLL into DPPDiv (Darriba et al. 2013). DPPDiv originally relied on the MrBayes 4.0 beta (from 2005) PLF implementation for carrying out likelihood evaluations on rooted trees. To minimize the coding effort and circumvent complicated and potentially error-prone modifications to the DPPDiv proposals, we designed a one-to-one mapping of the DPPDiv tree data structure onto the unrooted PLL tree data structure.

We obtained sequential speedups of 2.4–7.8 over the original implementation. By deploying fine-grain loop-level parallelism with PThreads, we obtained near-optimal parallel speedups on sufficiently large input datasets. Using the PThreads version on a 48-core shared memory system in conjunction with vector instructions, DPPDiv runtime was reduced by an overall factor of more than 100 (factor of 350 in the best case). A detailed description of the experimental setup and results can be found in (Darriba et al. 2013). Datasets are available at https://github.com/ddarriba/pll-dppdiv.

### IQ-TREE

We used PLL to re-implement the time-consuming NNI search in IQ-TREE. Integration with PLL took one of the authors of IQ-TREE < 1 one month of programming time. The new version is called ${\text{IQ-TREE}}_{PLL}$.

**Figure 3.**
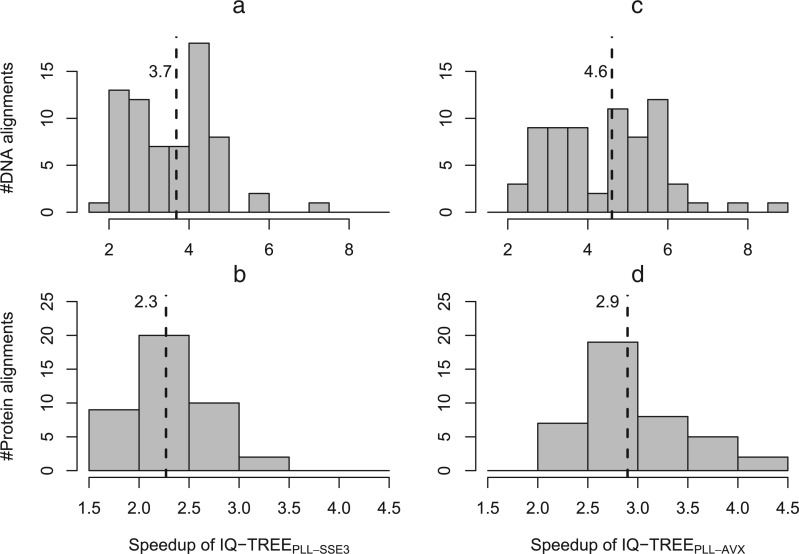
Speedups of ${\text{IQ-TREE}}_{PLL}$ compared with IQ-TREE for empirical DNA (a and c) and protein alignments (b and d). Two PLL versions were used, one with SSE3 (a, b) and the other with AVX (b, d) vector intrinsics. The dashed vertical lines indicate the median speedups.

We compared inference times of ${\text{IQ-TREE}}_{PLL}$ against IQ-TREE on empirical datasets from TreeBASE (http://www.treebase.org) on an Intel i5-2500K core. We downloaded 70 DNA alignments containing between 200 and 800 sequences (date accessed November 2012) and 47 protein alignments containing 50–400 sequences. TreeBASE matrix IDs are available in the Supplementary Material available on Dryad http://dx.doi.org/10.5061/dryad.9dk62. Figures 3a and b show the distribution of runtime ratios between IQ-TREE and ${\text{IQ-TREE}}_{PLL-SSE3}$ (i.e., the PLL is compiled with SSE3 instructions) for DNA and protein alignments, respectively. For DNA alignments ${\text{IQ-TREE}}_{PLL-SSE3}$ is between 1.8 and 7.0 times (median: 3.8) faster. For protein alignments the speedup ranges between 1.6 to 3.3 (median: 2.2). When using the AVX version of the PLL, the median speedup of ${\text{IQ-TREE}}_{PLL-AVX}$ on DNA data increases to 4.7 and to 2.9 on protein data (Fig. 3c and d).

For the sake of completeness, we also assessed the PThreads performance of ${\text{IQ-TREE}}_{PLL-SSE3}$ on a DNA (767 taxa, 5814 sites) and a protein (338 taxa, 1355 sites) dataset using 8 cores on an AMD Opteron 6132 processor. Note that, for the DNA dataset the time spent by ${\text{IQ-TREE}}_{PLL-SSE3}$ in the PLL, and hence the parallelized part of the code amounts to 95% of total runtime for DNA data and to 99% of total run time for protein data. Thus, the speedups are limited by Amdahl's law (1967). Taking into account the total execution time, we measured a total speedup of 2.62 (2.84 for the PLL kernel) for the DNA dataset and 5.64 (5.87) for the protein dataset. In general, the parallel scalability of protein likelihood calculations is better than for DNA data because one needs to conduct approximately $20^{2}$ instead of $4^{2}$ numerical operations per alignment site. Hence, more computational work is done per parallel region and the computation to communication ratio is more favorable.

## Conclusion

The PLL is a software library for developing high-performance likelihood-based phylogenetic software. It provides all necessary data structures and functions required to calculate the likelihood of a topology, explore the tree space, and perform model parameter optimization on partitioned datasets. The PLL is highly optimized, exploits state-of-the-art vector instructions (SSE3, AVX, AVX-2), and takes advantage of multi-core architectures, compute clusters, and the Intel Xeon PHI. By integrating PLL into DPPDiv, we obtained speedups of up to a factor of 7.8 on a single CPU and up to 350-fold speedups on a 48-core system. The integration with IQ-TREE yielded speedups of up to a factor of 7. Under double precision and with numerical scaling enabled, the sequential PLL version is 1.9 times faster than the BEAGLE library. The multi-threaded version of PLL is 3 times faster. Finally, the PLL requires ∼ 12% less memory than BEAGLE when scaling is enabled. Forthcoming extensions include an adaptation of the PLF for the AVX-512 instruction set, a low-level application programming interface that does not require a tree data structure, bindings for programming languages such as Python, and incorporating the new load balance algorithm into PLL.

## Supplementary Material

Data available from the Dryad Digital Repository: http://dx.doi.org/10.5061/dryad.9dk62.

## Funding

T.F. is supported by DFG project STA/860-4. F.I.-C. was supported by DFG projects STA/860-3 and STA/860-2. L.-T.N. and A.v.H. are supported by the University of Vienna (Initiativkolleg I059-N). B.Q.M. and A.v.H. are supported by the Austrian Science Fund–FWF (I760-B17).
